# Dental development in patients with agenesis

**DOI:** 10.1007/s00414-016-1450-0

**Published:** 2016-09-18

**Authors:** A. Lebbe, M. Cadenas de Llano-Pérula, P. Thevissen, A. Verdonck, S. Fieuws, G. Willems

**Affiliations:** 1 Department of Oral Health Sciences-Orthodontics, KU Leuven and Dentistry, University Hospitals Leuven, Kapucijnenvoer 7, 3000 Leuven, Belgium; 2 Department of Oral Health Sciences-Forensic Dentistry, KU Leuven and Dentistry, University Hospitals Leuven, Kapucijnenvoer 7, Leuven, 3000 Belgium; 3 Interuniversity Institute for Biostatistics and statistical Bioinformatics, KU Leuven and University Hasselt, Leuven, Belgium

**Keywords:** Forensic odontology, Agenesis, Dental development, Orthodontics

## Abstract

**Aim:**

Recent research concerning tooth development and dental agenesis suggests that specific genes are associated with agenesis, and that these genetic factors could also cause delayed dental development of the remaining teeth. The objective of this study was to evaluate whether dental development of patients with agenesis is delayed, compared to a control group.

**Subjects and method:**

Panoramic radiographs of 1145 patients with dental agenesis were collected (452 males, 693 females) aged 6.2 to 24.8 years. The control group included 2032 panoramic radiographs (977 males, 1055 females) aged 6.0 to 24.4 years. A total of 3177 orthopantomograms were staged according to Demirjian. All left permanent teeth present in the mandible (except third molars) were considered. In order to evaluate the difference between patients with and without agenesis, a developmental score (DS) was calculated. The association between the DS and the number of agenetic teeth was evaluated with a Spearman correlation.

**Results:**

Based on the DS, patients with agenesis have a delayed development compared to patients in the control group (*p* < 0.0001). Within the agenesis group, there is a weak relation between the number of agenetic teeth and the DS: the higher the number of teeth with agenesis, the lower the DS (*p* < 0.0001 and *p* = 0.06 for females and males, respectively).

**Conclusion:**

The obtained results can be an important factor for treatment planning in patients with dental agenesis. Moreover, the presence of agenesis needs to be taken into account when using age estimation methods based on permanent tooth development.

## Introduction

Dental agenesis or hypodontia is one of the most common anomalies in the development of the human dentition. It describes the absence of at least one tooth, excluding the third molars. In Europe, a prevalence is reported of 4.6 % in males and 6.3 % in females [1], being 1.4 times higher for females than for males. The mandibular second premolar is the most frequently affected tooth, followed by the maxillary lateral incisor and second premolar. The absence of maxillary central incisors, first molars and canines seems to be very rare. Anodontia refers to a complete absence of teeth, while oligodontia refers to the absence of six or more teeth, apart from the third molars, only seen in 2.6 % of patients with agenesis [1, 2]. In most patients with dental agenesis, there is only one or two teeth missing (48 and 35 % of the affected patients, respectively).

Several dentofacial anomalies are reported to be associated with agenesis. These include the following: reduction in tooth size or form, ectopic eruption of maxillary canines, infraposition of primary molars, taurodontism, enamel hypoplasia or hypocalcification. Agenesis can occur as a non-syndromic familial form, but can also be a part of a recognized clinical syndrome [3]. Online Mendelian Inheritance in Man lists 116 different syndromic conditions that include hypodontia as a part of their phenotypic spectrum of anomalies [4].

Agenesis also seems to be associated with delayed dental development [5–10]. Recent advances have suggested that specific genes, such as *PAX9*, *MSX1* and *AXIN2*, are associated with dental agenesis, and that these genetic factors could also cause delayed dental development of the remaining teeth [3, 7]. However, there is no consensus in the literature concerning this statement.

In order to study dental formation, many authors have described different techniques for the developmental staging of tooth formation [11–14]. The technique described by Demirjian is one of the most widely used developmental staging methods [11, 15].

A better understanding of the dental development in patients with agenesis is important not only for orthodontic diagnosis and treatment planning but also for age estimation of children with unknown birth data or for forensic purposes.

The objective of this study is to evaluate if dental development of patients with agenesis is delayed, compared to a control group.

## Material and methods

Selected were 1145 panoramic radiographs of patients with dental agenesis: 452 males (39.5 %) and 693 females (60.5 %), age range 6.2 to 24.8 years, mean age 12.0 years (SD 2.7 years). In Tables 1 and 2, an overview is given of the percentage of patients with a given number of teeth with agenesis and the percentage of patients with agenesis of a given tooth type.

**Table 1:** Descriptive information agenesis group—percentage of patients with a specific number of teeth with agenesis

Number of teeth with agenesis	Statistic	Males	Females
	*N*	452	693
	Mean	2.3	2.3
	Std	2.29	2.32
	Median	2.0	2.0
	IQR	(1.0; 2.0)	(1.0; 2.0)
	Range	(1.0; 21.0)	(1.0; 21.0)
Number of teeth with agenesis			
1	*n*/*N* (%)	187/452 (41.37 %)	285/693 (41.13 %)
2	*n*/*N* (%)	163/452 (36.06 %)	249/693 (35.93 %)
3	*n*/*N* (%)	35/452 (7.74 %)	60/693 (8.66 %)
4	*n*/*N* (%)	31/452 (6.86 %)	42/693 (6.06 %)
5	*n*/*N* (%)	10/452 (2.21 %)	14/693 (2.02 %)
6	*n*/*N* (%)	5/452 (1.11 %)	11/693 (1.59 %)
7	*n*/*N* (%)	4/452 (0.88 %)	6/693 (0.87 %)
8	*n*/*N* (%)	5/452 (1.11 %)	5/693 (0.72 %)
9	*n*/*N* (%)	2/452 (0.44 %)	3/693 (0.43 %)
10	*n*/*N* (%)	4/452 (0.88 %)	3/693 (0.43 %)
11	*n*/*N* (%)	1/452 (0.22 %)	2/693 (0.29 %)
12	*n*/*N* (%)	0/452 (0.00 %)	5/693 (0.72 %)
13	*n*/*N* (%)	0/452 (0.00 %)	2/693 (0.29 %)
15	*n*/*N* (%)	1/452 (0.22 %)	2/693 (0.29 %)
16	*n*/*N* (%)	2/452 (0.44 %)	1/693 (0.14 %)
17	*n*/*N* (%)	0/452 (0.00 %)	1/693 (0.14 %)
18	*n*/*N* (%)	1/452 (0.22 %)	0/693 (0.00 %)
19	*n*/*N* (%)	0/452 (0.00 %)	1/693 (0.14 %)
21	*n*/*N* (%)	1/452 (0.22 %)	1/693 (0.14 %)

**Table 2:** Descriptive information agenesis group—percentage of patients with/without agenesis of a specific tooth type

Tooth		Males (*n*/*N*)	Females (*n*/*N*)
17	NA	441/452 (97.57 %)	668/693 (96.39 %)
	A	11/452 (2.43 %)	25/693 (3.61 %)
16	NA	449/452 (99.34 %)	685/693 (98.85 %)
	A	3/452 (0.66 %)	8/693 (1.15 %)
15	NA	394/452 (87.17 %)	550/693 (79.37 %)
	A	58/452 (12.83 %)	143/693 (20.63 %)
14	NA	434/452 (96.02 %)	672/693 (96.97 %)
	A	18/452 (3.98 %)	21/693 (3.03 %)
13	NA	435/452 (96.24 %)	675/693 (97.40 %)
	A	17/452 (3.76 %)	18/693 (2.60 %)
12	NA	321/452 (71.02 %)	512/693 (73.88 %)
	A	131/452 (28.98 %)	181/693 (26.12 %)
11	NA	452/452 (100.00 %)	693/693 (100.00 %)
21	NA	452/452 (100.00 %)	693/693 (100.00 %)
22	NA	326/452 (72.12 %)	516/693 (74.46 %)
	A	126/452 (27.88 %)	177/693 (25.54 %)
23	NA	432/452 (95.58 %)	673/693 (97.11 %)
	A	20/452 (4.42 %)	20/693 (2.89 %)
24	NA	439/452 (97.12 %)	675/693 (97.40 %)
	A	13/452 (2.88 %)	18/693 (2.60 %)
25	NA	391/452 (86.50 %)	559/693 (80.66 %)
	A	61/452 (13.50 %)	134/693 (19.34 %)
26	NA	451/452 (99.78 %)	688/693 (99.28 %)
	A	1/452 (0.22 %)	5/693 (0.72 %)
27	NA	440/452 (97.35 %)	672/693 (96.97 %)
	A	12/452 (2.65 %)	21/693 (3.03 %)
47	NA	433/452 (95.80 %)	658/693 (94.95 %)
	A	19/452 (4.20 %)	35/693 (5.05 %)
46	NA	452/452 (100.00 %)	690/693 (99.57 %)
	A	0/452 (0.00 %)	3/693 (0.43 %)
45	NA	271/452 (59.96 %)	416/693 (60.03 %)
	A	181/452 (40.04 %)	277/693 (39.97 %)
44	NA	433/452 (95.80 %)	680/693 (98.12 %)
	A	19/452 (4.20 %)	13/693 (1.88 %)
43	NA	448/452 (99.12 %)	689/693 (99.42 %)
	A	4/452 (0.88 %)	4/693 (0.58 %)
42	NA	425/452 (94.03 %)	652/693 (94.08 %)
	A	27/452 (5.97 %)	41/693 (5.92 %)
41	NA	421/452 (93.14 %)	662/693 (95.53 %)
	A	31/452 (6.86 %)	31/693 (4.47 %)
31	NA	414/452 (91.59 %)	663/693 (95.67 %)
	A	38/452 (8.41 %)	30/693 (4.33 %)
32	NA	430/452 (95.13 %)	656/693 (94.66 %)
	A	22/452 (4.87 %)	37/693 (5.34 %)
33	NA	448/452 (99.12 %)	688/693 (99.28 %)
	A	4/452 (0.88 %)	5/693 (0.72 %)
34	NA	436/452 (96.46 %)	677/693 (97.69 %)
	A	16/452 (3.54 %)	16/693 (2.31 %)
35	NA	256/452 (56.64 %)	377/693 (54.40 %)
	A	196/452 (43.36 %)	316/693 (45.60 %)
36	NA	452/452 (100.00 %)	689/693 (99.42 %)
	A	0/452 (0.00 %)	4/693 (0.58 %)
37	NA	435/452 (96.24 %)	659/693 (95.09 %)
	A	17/452 (3.76 %)	34/693 (4.91 %)

*NA* no agenesis, *A* agenesis

**Fig. 1:**
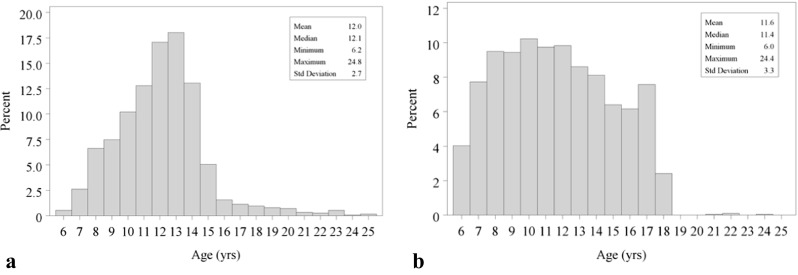
Descriptive information. **a** Number of patients with agenesis per age category. **b** Number of control patients per age category

In the control group, 2032 panoramic radiographs were included: 977 males (48.1 %) and 1055 female (51.9 %), age range 6.0 to 24.4 years, mean age 11.6 years (SD 3.3 years) (Fig. 1). This group was taken from a previous study by Willems et al. [16], which implies that the control and the agenesis group were not scored by the same observer. A total of 3177 orthopantomograms were staged according to Demirjian [11]. All left permanent teeth present in the mandible (excluding the third molar) were considered. If one tooth could not be visualized correctly due to i.e. image distortion, it was not scored. The staging method by Demirjian is a very clear and easy procedure. As a test, two observers staged all teeth in the third quadrant (except for the third molar) of 17 patients and repeated this procedure 2 weeks later.

### Inclusion criteria

The availability of a panoramic radiograph of adequate image quality was established as inclusion criterion, taken before the start of orthodontic treatment. The medical files of the patients were checked and those patients with a medical history that could affect the presence or development of the teeth were excluded from the study. For each patient, only one radiograph was selected. If more than one radiograph was available, the one chosen was from the age category of which, at that time, the smallest number of patients had been included.

### Statistical analysis

First, a continuation ratio (CR) model was used per tooth position to model the ordinal Demirjian stages [17]. The CR model can be considered as a set of binary logistic regression models. In the forward formulation of the CR model, each Demirjian stage is contrasted with a grouping of stages of following levels. The first binary logistic regression model then contrasts subjects with stage A with those with stage B or following stages. The second binary logistic regression model contrasts subjects with stage B with those with stage C or following stages in the dataset restricted to subjects with stages beyond stage A, and so on. Note that this model is referred to as a transition model by Boldsen et al. [18]. If the lowest Demirjian stages had a low prevalence, these were combined into a single category. Each logit in the CR model was modelled as a function of age and the parameters were allowed to differ between patients with and without agenesis. For example, in a CR model for Demirjian stages ≤C to H, there were 5 logits modelled as function of age and group. Hence, there were 10 parameters (5 intercepts, 5 slopes) describing the difference between both groups. A likelihood-ratio test was performed to evaluate if the groups differ in their relation between age and Demirjian stage. Two plots were constructed to visualize the difference between the groups. First, a plot with at each age the probability of each stage. Second, a plot with the transition probabilities at each age, i.e. the probability of developing from one stage to a following stage. From the CR model, the mean age at which the transition is made from a specific stage to the next stage is reported. Note that this age is in between the mean age of patients being in one stage and the mean age of patients being in the next stage.

In order to evaluate the difference between patients with and without agenesis, and to verify the relation between the speed of development and the number of agenesis, an index was constructed to quantify, at patient level, the degree of development, preserving the ordinal character of the Demirjian stages and handling the presence of missing values. Using a multivariate version of the CR model, such an index has been constructed analogously as in Thevissen et al. [19] and is referred to as the developmental score (DS), which is a normal distributed variable (*z* score) with mean and standard deviation equal to 0 and 1, respectively. A DS equal to zero corresponds to a patient with an average development level for his/her age in the current study. The distribution of the DS was compared between patients with and without agenesis using a Mann-Whitney U test. The area under the curve (AUC) was reported, which reflects the degree of discriminative ability (0.5 = no discrimination, 1 = perfect discrimination). The association between the DS and the number of agenetic teeth was evaluated with a Spearman correlation.

In summary, we did not estimate age of the patients in the control and the agenesis group, we staged all teeth present in the left mandible and compared dental development between the two groups.

All analyses have been performed using SAS software, version 9.2 of the SAS System for Windows. Copyright © 2002 SAS Institute Inc. SAS and all other SAS Institute Inc. product or service names are registered trademarks or trademarks of SAS Institute Inc., Cary, NC, USA.

## Results

Inter and intra-observer comparison revealed a great similarity in scoring. Inter-observer reliability (calculated over the 7 teeth) was set on 0.94 and 0.87 at the first and second replication, respectively, while intra-observer equaled 0.95 and 0.96. When a different stage was found, the difference was never more than one stage.

**Fig. 2:**
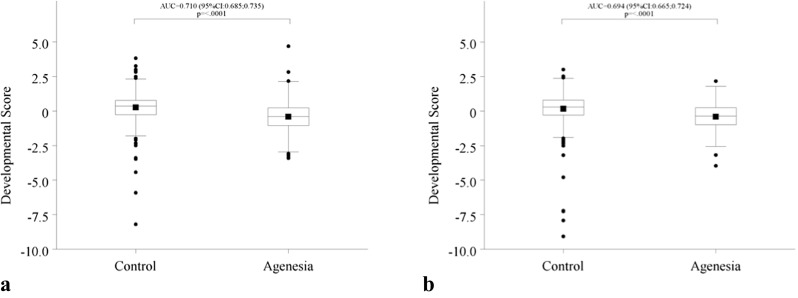
Comparison developmental score between groups. **a** Female. **b** Male

Based on the DS, patients with agenesis have a delayed tooth development compared to patients in the control group. The difference equals 0.68 standard deviations (note that the standard deviation of the DS equals 1 by definition) for females (AUC = 0.710 (CI 0.685 to 0.735), *p* < 0.0001) and 0.58 standard deviations for males (AUC = 0.694 (CI 0.665 to 0.724), *p* < 0.0001) (Fig. 2).

**Fig. 3:**
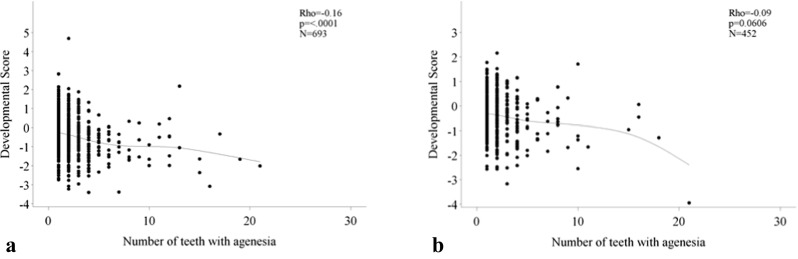
Relation developmental score with number of agenetic teeth. **a** Female. **b** Male

Within the group of patients with agenesis, there is a weak relation between the number of agenetic teeth and the DS: the higher the number of teeth with agenesis, the lower the DS: rho = −0.16 (*p* < 0.0001) and rho = −0.09 (*p* = 0.06) for females and males, respectively (Fig. 3).

**Fig. 4:**
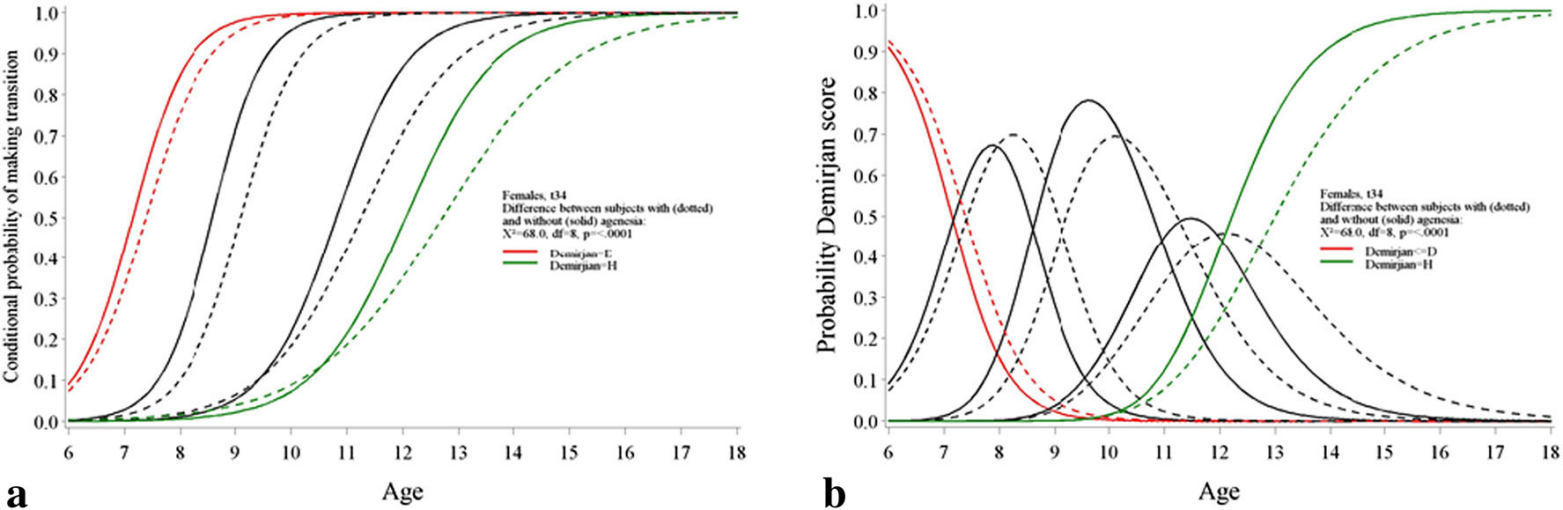
Comparison control-agenesis using a continuation-ratio model. The example refers to tooth t34 for female patients. **a** Conditional probability of making the transition from the lower stage to the specific stage for tooth t34 in female patients. For example, the *green line* refers to the probability of going from stage G to stage H. The *red line* refers to the probability of going from a stage before E to stage E. The *dotted line* stands for the agenesis group, the *solid line* refers to the control group. **b** At each age, the probability of each Demirjian score. For example, the *green line* refers to the probability of being at stage H at a certain age, for tooth 34 in females. The *red line* refers to the probability of being at stage D or earlier at a certain age. Again, the *dotted line* stands for the agenesis group, the *solid line* refers to the control group

The result on the delayed development based on the DS is confirmed by the results of the continuation-ratio model applied per tooth position. For females as well as for males, the timing of tooth development differs significantly between patients with agenesis and patients in the control group in all positions, except for tooth 31 in female subjects (Table 3). The probability to go from one stage to a next stage is lower at each specific age for patients with agenesis compared to patients in the control group. As an illustration, the probability curves were presented for tooth 34 in female subjects in Fig. 4.

**Table 3:** Results likelihood-ratio test (LRT) comparing patients with and without agenesis using a continuation-ratio model per gender and per tooth position

Gender	Position	Chi^2^	*df*	*p* value
Females	31	9.1	6	0.1671
	32	17.5	6	0.0075
	33	33.3	8	<0.0001
	34	68.0	8	<0.0001
	35	116.8	10	<0.0001
	36	69.9	4	<0.0001
	37	258.6	10	<0.0001
Males	31	14.0	6	0.0300
	32	19.7	6	0.0031
	33	55.1	8	<0.0001
	34	57.4	10	<0.0001
	35	101.7	10	<0.0001
	36	37.9	4	<0.0001
	37	149.5	10	<0.0001

*Df* degrees of freedom, *Chi* ^*2*^ chi-square statistic of LRT

In order to clearly represent the clinical differences between the two groups, both the age at transition to a certain stage and the point prediction of the age in a specific stage were reported for each tooth position and gender (Table 4).

**Table 4:** Overview of age at transition to a specific stage and point predictions for age in a certain stage

		Number of patients		Age at transition to stage (95 % CI)		Point predictions age in stage (95 % CI)	
Tooth	Demirjian	Control	Agenesis	Control	Agenesis	Control	Agenesis
Males							
31	E	30	.			6 (6.0; 9.7)	6 (6.0; 7.4)
31	F	72	6	5.3 (2.1; 8.5)	5.0 (0.2; 1.3)	6.1 (6.0; 8.2)	6 (6.0; 8.4)
31	G	115	37	7.1 (5.3; 8.8)	6.6 (4.3; 9.0)	7.8 (6.0; 10.5)	7.7 (6.0; 10.0)
31	H	754	346	8.2 (5.1; 11.3)	8.8 (6.5; 11.1)	18 (8.5; 18.0)	18 (8.9; 18.0)
32	E	64	4			6 (6.0; 8.7)	6 (6.0; 7.4)
32	F	107	26	6.6 (4.3; 8.9)	7.0 (6.0; 7.9)	7.3 (6.0; 9.3)	7.6 (6.4; 9.4)
32	G	153	61	7.9 (5.8; 10.1)	8.4 (6.9; 9.9)	8.8 (6.7; 11.8)	9.2 (7.4; 12.7)
32	H	640	301	9.5 (6.5; 12.5)	9.9 (6.2; 13.6)	18 (9.5; 18.0)	18 (9.7; 18.0)
33	D	79	12			6 (6.0; 8.8)	6 (6.0; 8.4)
33	E	120	50	6.8 (4.5; 9.1)	7.3 (5.3; 9.2)	7.5 (6.0; 9.3)	8.2 (6.2; 10.3)
33	F	311	143	8.3 (6.4; 10.1)	9.1 (7.3; 11.0)	9.9 (7.5; 12.8)	10.4 (8.1; 13.5)
33	G	148	109	11.6 (9.3; 13.9)	11.9 (9.3; 14.6)	12.4 (9.9; 15.4)	13 (10.3; 17.4)
33	H	310	125	13.1 (10.2; 16.1)	13.3 (7.6; 19.1)	18 (12.7; 18.0)	18 (12.4; 18.0)
34	C	23	2			6 (6.0; 9.8)	6 (6.0; 8.4)
34	D	135	27	4.2 (0; 8.6)	4.7 (1.5; 7.9)	6.4 (6.0; 8.8)	6.5 (6.0; 9.4)
34	E	120	52	7.8 (5.8; 9.7)	7.9 (5.4; 10.5)	8.4 (6.5; 10.6)	8.8 (6.4; 10.7)
34	F	259	127	9.0 (6.8; 11.1)	9.6 (7.8; 11.4)	10.4 (8.1; 13.1)	10.7 (8.6; 13.9)
34	G	98	104	11.8 (9.5; 14.1)	12.0 (9.0; 15.0)	12.3 (10.0; 15.1)	13 (10.3; 17.3)
34	H	340	121	12.5 (9.6; 15.4)	13.3 (7.6; 19.1)	18 (12.5; 18.0)	18 (12.4; 18.0)
35	C	53	18			6 (6.0; 9.5)	6 (6.0; 10.1)
35	D	149	22	6.0 (2.7; 9.3)	7.6 (3.9; 11.2)	7.2 (6.0; 9.5)	8.1 (6.0; 11.0)
35	E	125	44	8.3 (6.2; 10.5)	8.7 (5.4; 11.9)	9 (6.8; 11.7)	9.9 (7.2; 12.6)
35	F	264	88	9.4 (6.7; 12.1)	10.7 (8.1; 13.3)	11 (8.4; 14.2)	12 (9.4; 16.3)
35	G	111	51	12.5 (9.8; 15.3)	13.2 (8.6; 17.8)	13.1 (10.4; 16.3)	14.3 (11.4; 17.9)
35	H	264	30	13.4 (10.0; 16.9)	14.9 (10.4; 19.4)	18 (13.1; 18.0)	18 (13.5; 18.0)
36	F	98	25			6 (6.0; 9.0)	6 (6.0; 9.0)
36	G	224	104	7.0 (4.6; 9.5)	7.8 (5.5; 10.1)	8.3 (6.0; 11.0)	9 (6.4; 12.5)
36	H	647	318	9.6 (6.7; 12.4)	10.4 (7.1; 13.7)	18 (9.4; 18.0)	18 (10.0; 18.0)
37	C	67	12			6 (6.0; 9.5)	6 (6.0; 10.6)
37	D	164	64	6.3 (3.2; 9.5)	6.2 (2.2; 10.1)	7.6 (6.0; 9.4)	7.9 (6.0; 10.7)
37	E	159	69	8.6 (6.9; 10.4)	9.4 (6.7; 12.2)	9.5 (7.5; 12.1)	10.2 (7.5; 13.1)
37	F	186	165	10.2 (7.7; 12.7)	10.8 (7.9; 13.7)	11.3 (8.9; 13.9)	12.3 (9.6; 15.5)
37	G	195	93	12.2 (9.7; 14.7)	13.5 (10.7; 16.4)	13.5 (11.0; 16.4)	14.8 (12.1; 17.7)
37	H	197	27	14.8 (12.0; 17.5)	15.9 (13.3; 18.6)	18 (13.9; 18.0)	18 (14.7; 18.0)
Females							
31	E	10	1			6 (6.0; 8.5)	6 (6.0; 8.9)
31	F	45	8	5.9 (4.7; 7.0)	5.9 (4.9; 6.9)	6 (6.0; 8.1)	6 (6.0; 8.9)
31	G	103	42	6.5 (4.5; 8.5)	5.6 (2.4; 8.8)	7.2 (6.0; 9.1)	6.9 (6.0; 9.8)
31	H	894	555	7.8 (5.7; 9.9)	7.8 (4.9; 10.7)	18 (8.1; 18.0)	18 (8.0; 18.0)
32	E	36	6			6 (6.0; 8.1)	6 (6.0; 7.9)
32	F	82	27	6.3 (4.5; 8.1)	6.3 (4.5; 8.1)	6.9 (6.0; 8.2)	6.9 (6.0; 8.9)
32	G	138	75	7.5 (6.2; 8.8)	7.5 (5.4; 9.6)	8.2 (6.7; 10.8)	8.4 (6.4; 11.6)
32	H	794	483	8.7 (6.1; 11.4)	9.1 (5.7; 12.4)	18 (8.9; 18.0)	18 (9.1; 18.0)
33	D	36	3			6 (6.0; 8.4)	6(6.0; 8.1)
33	E	93	45	6.0 (3.7; 8.4)	5.4 (3.1; 7.8)	6.8 (6.0; 8.4)	6.7 (6.0; 9.2)
33	F	261	152	7.5 (5.9; 9.2)	7.9 (5.7; 10.1)	8.8 (6.8; 11.3)	9.1 (6.6; 12.0)
33	G	174	163	10.2 (8.3; 12.2)	10.4 (7.7; 13.1)	11 (8.7; 14.8)	11.4 (8.6; 16.1)
33	H	488	296	11.6 (7.6; 15.5)	11.7 (6.1; 17.3)	18 (11.2; 18.0)	18 (11.1; 18.0)
34	D	88	28			6 (6.0; 8.3)	6 (6.0; 8.7)
34	E	138	77	7.1 (5.4; 8.9)	7.4 (5.4; 9.3)	7.9 (6.1; 9.5)	8.2 (6.1; 10.2)
34	F	226	175	8.6 (7.0; 10.2)	9.1 (7.3; 10.9)	9.6 (7.8; 12.2)	10.1 (8.0; 13.3)
34	G	148	165	10.8 (8.6; 13.1)	11.3 (8.3; 14.2)	11.5 (9.2; 14.3)	12.1 (9.4; 15.9)
34	H	448	217	12.0 (9.2; 14.9)	12.7 (8.6; 16.8)	18 (11.9; 18.0)	18 (12.1; 18.0)
35	C	35	15			6 (6.0; 9.4)	6 (6.0; 9.6)
35	D	138	28	5.4 (1.7; 9.1)	6.7 (2.9; 10.6)	6.8 (6.0; 9.0)	7.9 (6.0; 10.3)
35	E	107	47	8.0 (5.9; 10.0)	8.4 (5.7; 11.1)	8.5 (6.5; 10.9)	9.3 (6.9; 12.7)
35	F	269	138	8.8 (6.4; 11.3)	9.6 (6.0; 13.1)	10.3 (8.0; 13.3)	11.3 (8.4; 15.0)
35	G	156	77	11.7 (9.2; 14.2)	12.7 (9.2; 16.2)	12.5 (9.9; 15.7)	13.8 (10.9; 17.6)
35	H	345	58	13.1 (9.8; 16.5)	14.4 (9.8; 19.0)	18 (12.7; 18.0)	18 (13.2; 18.0)
36	F	49	26			6 (6.0; 8.1)	6 (6.0; 8.8)
36	G	221	140	6.6 (4.9; 8.2)	7.1 (4.6; 9.6)	7.7 (6.0; 10.1)	8.3 (6.0; 11.8)
36	H	782	520	9.0 (6.7; 11.3)	9.7 (6.1; 13.3)	18 (9.1; 18.0)	18 (9.3; 18.0)
37	C	39	20			6 (6.0; 8.5)	6 (6.0; 9.9)
37	D	157	77	6.2 (4.0; 8.4)	6.2 (2.5; 10.0)	7.2 (6.0; 9.2)	7.5 (6.0; 10.5)
37	E	149	123	8.3 (6.3; 10.2)	8.7 (5.8; 11.7)	9 (7.0; 11.3)	9.7 (6.8; 13.2)
37	F	190	235	9.7 (7.4; 11.9)	10.3 (6.7; 14.0)	10.6 (8.5; 13.1)	11.8 (8.8; 15.6)
37	G	248	141	11.5 (9.1; 13.9)	13.1 (9.3; 16.9)	12.9 (10.3; 16.1)	14.6 (11.7; 18.0)
37	H	267	59	14.3 (11.3; 17.3)	16.0 (11.0; 20.9)	18 (13.4; 18.0)	18 (13.8; 18.0)

## Discussion

### Overview of the existing literature

A literature search for dental development in patients with agenesis revealed several studies, each of them with a different study design, study group and staging technique (Table 5). All of these studies detected a delay in the agenetic group, but each of them quantified it differently, making fair comparisons impossible. It also seems that the choice of the staging technique is determining for the extent of the results.

**Table 5:** Overview of the related literature

Author	Staging technique	Delay in dental development?	Degree of delay
Tunç et al. [9]	Demirjian	Yes	Mean difference of the estimated dental age of 0.3 years between the agenesis and the control group
Rune and Sarnas [8]	Haavikko	Yes	1.8 years for boys, 2.0 years for girls, compared with the reference tables from Haavikko
Uslenghi et al. [10]	Haavikko	Yes	Mean delay of 1.51 years of dental age in the agenesis group compared to the control group
Daugaard et al. [5]	Haavikko	Yes	Not quantified
Ruiz-Mealin et al. [7]	Haavikko	Yes	Delay in dental age of 1.2 years in the agenesis group
	Demirjian	Yes	Delay in dental age of 1.64 years in the agenesis group
Gelbrich et al. [6]	Nolla	Yes	Dental development was 8.6 months delayed in subjects with missing second premolars
Odagami et al. [27]	Moorrees	Yes, but not significant	Delay in dental development of 3 months

### Staging method

In order to study dental development, many authors have described different techniques for the staging of tooth formation, where dental maturation is arbitrarily divided into successive developmental stages. The thresholds between the stages need to be well described and refer to observable anatomical tooth traits. The number and the duration of the stages differ depending on the specific technique: the more stages a technique involves, the less precise the classification, because of the difficulty of reliably identifying the stages [20, 21]. For the purpose of age estimation in third molars, Thevissen et al. [22] recommended a staging method over complicated dimension measurements (length or width), or ratio calculations of these measurements. Previous studies [23] reported that the eight-stage technique described by Demirjian [11] performed best for observer agreement and provided the highest correlations between estimated and true age. It also classifies the different tooth developmental stages on the basis of objective observations thus avoiding the necessity to include tooth measurements. The reliability of age estimation methods based on the development of permanent teeth, except for the third molars, is reported with a 95 % confidence interval from ±0.65 for early tooth stages to ±2.59 years for late tooth stages [24].

### Diagnosis of agenesis

Diagnosis of dental agenesis is based on interpretation of a panoramic radiograph, combined with clinical examination [2]. Moorrees et al. [14] investigated the age of formation of 10 permanent teeth, finding that initial crown formation in the mandible occurred at the age of 6 months, 1.8, 3.0 and 3.5 years for the permanent canine, first premolar, second premolar and second molar, respectively. Initial crown formation of the first molar occurs before birth. More recently, Nyström et al. [25] found similar results, when assessing 2795 radiographs of 1970 Finns from birth to age 25. It is also stated that all permanent tooth crowns except the third molar have begun their mineralization by the age of 6 [2, 9]. Therefore, in this study, we excluded patients younger than 6 years. The radiographs were selected from an orthodontic patient group, so most of them were followed up longitudinally by the attending orthodontist. This factor tended to ensure that the diagnosis of dental agenesis was made even more secure. Unfortunately, very late development of the mandibular second premolar has been reported in a few cases. To avoid a false-positive diagnosis of agenesis of this tooth, the method proposed by Sharma et al. was used [26]. They suggest that the second premolar in the mandible is highly unlikely to develop if the adjacent first premolar is beyond stage ‘crown complete’ and the first molar is beyond stage ‘root one half’. This was checked in our agenesis group. Ten patients did not meet these requirements and their dental files were checked for radiographs at an older age to confirm the agenesis. The three cases, where there was no longitudinal follow-up confirmation, were excluded.

### Differences between gender or tooth position?

It was decided not to evaluate whether the results obtained depended on the specific tooth type or tooth position of the agenetic tooth. In addition, it was decided not to carry out statistical analyses to compare the dental development between genders. Table 4 contains some information regarding the differences in dental development based on tooth type or sex. For example, in almost all tooth positions, a faster dental development for girls was noted. For tooth 35, the estimated age where girls without agenesis reach stage G is 11.7 and 12.5 years for boys. However, these observations were not tested formally because this was not the aim of the study.

A difference in developmental delay between genders was noted. In males, the Spearman correlation was less clear in the relation between the number of agenetic teeth and the delay in dental development. This may be explained by the decreased number of males with a greater number of agenetic teeth included in our study group.

### Limitations

A high variability in the number of patients in each age category between groups was observed. In the agenesis group, a distinct peak around 12 years of age was detected; after 15 years of age, only a limited number of patients were included in each age category (Fig. 1). This can be explained by the sampling method. If a patient is diagnosed with dental agenesis, very often orthodontic treatment is required. Patients who had already experienced previous orthodontic treatment, possibly affecting dental development, were excluded. As such, the number of patients above 15 years of age was limited. In the control group, a more equal distribution of patients in each age category could be established due to a selective retrospective search in clinical databases of ‘normal’ children.

In Table 4, the point predictions of age in a stage are always ‘6 years’ in the lowest stage and ‘18 years’ in the highest stage, because they are based on the Bayes’ rule, where a uniform prior distribution is specified from 6 to 18 years. Therefore, the age estimations in the outer stages are always the extremes of the used prior distribution and clinical use of these outer stages is not recommended. Furthermore, in Table 4, age estimations are given for the later stages, but not for the earliest stages, especially not in those tooth types who start developing very early. To overcome these problems, the study group should be extended with more patients included in the lower and higher age categories and even lower age categories should also be included. However, diagnosis of agenesis is uncertain at ages lower than 6 years and at these young ages a panoramic radiograph is not often taken.

For tooth 31 in female subjects, this study did not find a significant difference in dental development between subjects with and without agenesis (Table 3). This could be explained by the lower age truncation of the sampled subjects, set at 6 years. The development of tooth 31 occurs very early. According to the study of Willems et al. [16], mean age in stage E (start root formation) is 4.49 and 4.61 years and mean age in stage H (apex closed) is 7.92 and 8.52 for girls and boys, respectively. Moreover, the image of a panoramic radiograph is often distorted in the developmental zone of the central incisors, due to overlap with the cervical vertebrae. When this was the case, the tooth was not staged in our study. As a consequence, there is less information about this tooth to capture possible differences.

### Clinical applicability and future remarks

In Table 4, an overview is given with the age at transition to a certain stage for each tooth and stage, and a point prediction of the age in a certain stage per tooth. By definition, the age at transition to a stage is lower than the predicted age for a patient being in this stage. Point predictions of age in a stage give an indication of how old a person should be if this patient presents with a specific tooth type in a certain stage. However, the accuracy of these data still needs to be tested in future research. This can be done by using these new tables for estimating the age of patients with agenesis, comparing the estimated dental age with the chronological age. The less difference between estimated and true age, the more accurate the new tables would be.

These adapted tables could be used for forensic age estimation, which is often requested to determine whether a patient is still a minor or not, leading to several legal consequences. In the case of a patient with dental agenesis, using the existing reference tables for normal patients, this person could mistakenly be considered to be a minor.

However, there is, in fact, a difference in the age estimation of patients with and without agenesis. In the latter, all teeth in the left mandible (except for the third molars) are staged. Using the reference tables of Willems et al. [16], summing up the scores from all seven teeth, directly results in the estimated dental age. For age estimation of patients with agenesis, this is not possible, since not all teeth are available for staging. In these patients, it would be better to concentrate on staging the latest developing tooth present in the left mandible, and this can give an estimation of age. Of course, this technique has some limitations with older patients who present with, for example, agenesis of the second premolar or the second molar in the left mandible, since all other available teeth could possibly already have completed dental development.

In future research, it could be interesting to explore whether there is a difference in dental development depending on tooth type or tooth position of the agenetic tooth.

Finally, the delay in dental development in patients with agenesis is also an important factor for orthodontic treatment planning as the age for onset of treatment and the duration of treatment depend on dental maturity.

## Conclusion

Dental development in patients with dental agenesis is delayed compared to patients in the control group (*p* < 0.0001). A weak correlation between the number of agenetic teeth and the degree of delay in dental development was observed (*p* < 0.0001 for females and *p* = 0.06 for males). These findings can have an impact on the orthodontic treatment planning and the dental age estimation outcomes of patients with agenesis.
